# Development of an Alfalfa SNP Array and Its Use to Evaluate Patterns of Population Structure and Linkage Disequilibrium

**DOI:** 10.1371/journal.pone.0084329

**Published:** 2014-01-09

**Authors:** Xuehui Li, Yuanhong Han, Yanling Wei, Ananta Acharya, Andrew D. Farmer, Julie Ho, Maria J. Monteros, E. Charles Brummer

**Affiliations:** 1 Forage Improvement Division, The Samuel Roberts Noble Foundation, Ardmore, Oklahoma, United States of America; 2 National Center for Genome Resources, Santa Fe, New Mexico, United States of America; 3 Forage Genetics International, Davis, California, United States of America; Nanjing Forestry University, China

## Abstract

A large set of genome-wide markers and a high-throughput genotyping platform can facilitate the genetic dissection of complex traits and accelerate molecular breeding applications. Previously, we identified about 0.9 million SNP markers by sequencing transcriptomes of 27 diverse alfalfa genotypes. From this SNP set, we developed an Illumina Infinium array containing 9,277 SNPs. Using this array, we genotyped 280 diverse alfalfa genotypes and several genotypes from related species. About 81% (7,476) of the SNPs met the criteria for quality control and showed polymorphisms. The alfalfa SNP array also showed a high level of transferability for several closely related *Medicago* species. Principal component analysis and model-based clustering showed clear population structure corresponding to subspecies and ploidy levels. Within cultivated tetraploid alfalfa, genotypes from dormant and nondormant cultivars were largely assigned to different clusters; genotypes from semidormant cultivars were split between the groups. The extent of linkage disequilibrium (LD) across all genotypes rapidly decayed to 26 Kbp at r^2^ = 0.2, but the rate varied across ploidy levels and subspecies. A high level of consistency in LD was found between and within the two subpopulations of cultivated dormant and nondormant alfalfa suggesting that genome-wide association studies (GWAS) and genomic selection (GS) could be conducted using alfalfa genotypes from throughout the fall dormancy spectrum. However, the relatively low LD levels would require a large number of markers to fully saturate the genome.

## Introduction

Alfalfa (*M. sativa* L.) is an important forage legume throughout the world. Alfalfa provides high nutrition animal feeds and as a perennial crop, offers numerous environmental benefits. The deep root system stabilizes soil and increases soil fertility through symbiotic biological nitrogen fixation, providing all the nitrogen needed for the alfalfa crop and a subsequent grain crop.Most alfalfa cultivars are synthetic populations developed by recurrent phenotypic selection. Although important agronomic traits such as winter survival and disease resistance have been improved by phenotypic selection, multiple-year phenotypic evaluations per cycle of selection limit rate of genetic gain. Partially due to the long breeding cycle, the improvement of biomass yield in alfalfa has been much slower compared to that in annual crops [1]. Minimal genetic improvement of biomass yield in alfalfa was found when comparing cultivars released from different eras [2]. Developing cultivars with high yield that are also tolerant to abiotic and biotic stresses is crucial to enhance alfalfa productivity and competitiveness in the world market.

Molecular markers can be used to dissect the genetic control of quantitative traits and predict breeding values of individuals. Indirect selection based on molecular markers can potentially reduce time per breeding cycle and accelerate alfalfa improvement. The genetic dissection of agronomic traits in alfalfa with molecular markers began in the 1990's, and to date QTLs have been identified in biparental populations for aluminum tolerance [3], biomass yield [4], persistence [5], winter hardiness [6], [7], and disease resistance [8]. However, due to large linkage blocks in these populations, the QTL were not localized to small regions of the chromosomes. Further fine mapping and validation of the alfalfa QTLs are costly and time consuming and thus have not been pursued in most research programs. Genome-wide association studies (GWAS) can be used to more precisely localize QTLs by evaluating historical recombination. Using GWAS, causal genes of agronomic traits have been successfully identified in various crops [9], [10], which can be used to efficiently improve traits influenced by a few genes via marker-assisted selection (MAS) [11]. For complex traits controlled by many genes with small effects, practical application is constrained by poor predictions based on a few major loci [12]. In contrast to pyramiding a few major QTLs via marker-assisted selection (MAS), breeders can apply genomic selection (GS), which uses whole-genome markers to predict the breeding values of individuals and thus can capture the effects from both major and minor QTLs [13]. Studies based on both simulated and experimental data suggested that GS improved prediction of breeding values compared to MAS for complex traits [14]–[16].

A large set of genome-wide markers able to be assayed with a high-throughput genotyping platform is required to perform GWAS and GS. Advances in next generation sequencing and bioinformatics enable detection of many SNP inexpensively, even for orphan crops without a sequenced reference genome. Using 454 sequencing, approximately 11,000 SNPs were discovered by sequencing two alfalfa genotypes contrasting for lignin and cell wall composition [17] and 40,000 SNPs were identified for two genotypes with varying responses to drought stress [18]. By sequencing transcriptomes of 27 diverse alfalfa genotypes, including wild diploids and cultivated tetraploids, we identified 900,000 SNPs [19]. Some of these SNP have been developed into markers using high resolution melting (HRM) [18], and these have been used to improve the resolution of linkage maps and to map candidate genes for traits such as cold tolerance and flowering time (Li et al., unpublished data). However, a high-throughput genotyping platform for alfalfa is still not available.

The experimental design of GWAS and GS can be optimized by accounting for the patterns of population structure and linkage disequilibrium (LD) in the populations employed. Proper GWAS statistical models need to be used to minimize false positive association caused by the underlying population structure [20]. Clear population structure corresponding to the subsp. *falcata* and subsp. *sativa* has been commonly found in alfalfa germplasm [21], [22], although less clear distinctions have been identified within cultivated germplasm. The number of markers required for a successful GWAS or GS project depends on the extent of LD in the population. In alfalfa, LD of 0.5 Mbp (p<0.01) was found in a breeding population with a relatively narrow genetic background using SSR markers, although the high mutation rate of SSR markers and the small sample size likely overestimated the LD [23]. Rapid LD decay was observed within candidate genes related to lignin biosynthesis in wild diploid alfalfa collections [24] and flowering time in ten tetraploid cultivars [25]. To facilitate GWAS and GS in alfalfa, evaluation of the genome-wide LD pattern using a large set of markers is needed.

In this study, our objectives were as follows: (a) to develop a high density SNP array that would provide a high-throughput genotyping platform for alfalfa; and (b) to assess patterns of population structure and LD in diverse alfalfa accessions that could guide appropriate experimental designs for subsequent GWAS and GS projects.

## Materials and Methods

### SNP detection, filtering, and selection

We previously identified about 900,000 SNPs by transcriptome sequencing 27 diverse alfalfa genotypes [19]. These ESTs and SNPs are publicly available at the Legume Information System (LIS, http://medsa.comparative-legumes.org/). From this set, we developed an Illumina Infinium array consisting of 10,441 SNPs. We identified SNP loci for the array as follows. First, only loci that had at least 10 reads in at least 20 of the 27 sequenced genotypes were retained; this resulted in about 400,000 SNPs. Second, we computed assay designing tool (ADT) scores and GoldenGate scores (http://support.illumina.com/array/array_software/assay_design_tool.ilmn) for these SNPs and about 180,000 having scores for both criteria that were greater than 0.4 were used for further selection. Third, from the literature, we identified candidate genes for aluminum tolerance, cell cycle, cell size, cell wall composition, drought tolerance, flowering time, growth rate, lignin biosynthesis, components of glandular trichomes, and winter hardiness. For sequences of the candidate genes identified in other plant species, we searched the alfalfa transcriptome sequences [19] for homologous sequences using BLAST, and the best hits with an E-value lower than 1×10^−5^ were selected. Up to three SNPs were selected from a given EST. In total, 6,468 SNPs from 2,640 candidate genes were included on the array (Table S1). Of the 6,468 SNPs, 3,241 aligned on the eight chromosomes of *M. truncatula* pseudomolecule v3.5. Lastly, in order to improve the genome coverage, we added 2,712 SNPs to fill the gaps between candidate gene SNPs. We also selected 1,261 SNPs from 552 ESTs that did not align to the eight chromosomes of *M. truncatula* pseudomolecule v3.5, assuming that some of them might be derived from genes specifically expressed in alfalfa.

### SNP array evaluation

We assayed 280 genotypes including 233 cultivated tetraploid subsp. *sativa*, one tetraploid subsp. *falcata*, 25 diploid subsp. *caerulea*, 15 diploid subsp. *falcata* and six genotypes from related species (Table S2) with this SNP array. The 233 tetraploid subsp. *sativa* genotypes spanned the fall dormancy spectrum of alfalfa germplasm and included multiple genotypes from the eleven fall dormancy standard check cultivars [26] and the four winter survival standard checks cultivars [27] (Table S2). The alfalfa entries also included 11 F_1_ progenies from bi-parental mapping populations (10 F_1_ progenies from the DM3 × DM5 population (a biparental population with broad fall dormancy variation (X. Li and E.C. Brummer, unpub. results) and one F_1_ progeny from the Altet4 × NECS-141 population [3]). In addition, we included genotypes of Bulldog 805, a cultivar adapted to the southeastern Coastal Plain of the USA and of four non-dormant populations (ND1, ND2, ND3, and ND4) selected for grazing tolerance using beef cattle in Tifton, GA. As part of our ongoing cultivar development program, we recently developed four new breeding populations by hand crossing 15 genotypes from each of the four ND populations with a different set of 15 genotypes from the cultivar Bulldog 805. The resulting populations were BR1 = ND1×Bulldog 805, BR2 = ND2×Bulldog 805, BR3 = ND3×Bulldog 805, and BR4 = ND4×Bulldog 805. We are interested in the extent of linkage disequilibrium in these populations. To estimate LD in these breeding populations, we used the genotypic data from the parental genotypes to infer the levels of LD in these populations.

For each genotype, fresh leaf tissue was used for DNA extraction using the Wizard® Genomic DNA Purification Kit (Promega, Cat # A1125) following the manufacturer's protocol. The purified DNA was quantified with the Quant-iT PicoGreen dsDNA Reagent and Kits (Invitrogen, Cat # P7589) and normalized to 55 ng/µl. Genotyping was performed on an Illumina iScan system at the National Center for Genome research (NCGR, Santa Fe, NM) following the manufacturer's protocol for the Infinium assay. The resulting intensity data were used to score SNPs with the Genotyping Module of the GenomeStudio software (Illumina Inc., San Diego CA) using a GenCall threshold of 0.15. For a given SNP marker, three clusters (AA, AB, and BB) could exist in diploid alfalfa but up to five clusters (AAAA, AAAB, AABB, ABBB, and BBBB) could be present in tetraploid alfalfa. In this study, 40 diploid alfalfa genotypes were scored at the same time as tetraploid alfalfa. The automated cluster algorithm with a cluster number of three was used to generate the initial calls. The initial SNP clusters were manually edited to refine the cluster position. For the SNPs in which five clusters could be clearly distinguished, manual editing was performed following the polyploidy protocol in GenomeStudio (Illumina Inc.). The diploid genotypes (AA, BB and AB) were useful to determine the tetraploid homozygous classes (AAAA and BBBB) and the duplex heterozygous class (AABB). To simplify analysis of population structure and LD, both diploid and tetraploid genotypes were scored with three classes (AA, AB and BB), where all three heterozygous classes (AAAB, AABB and ABBB) in tetraploid individuals were scored as heterozygous AB.

### Population structure

Each allele of a SNP marker was scored as 1 (presence) or 0 (absence). For a given SNP marker, missing values were first replaced by the mean value for the marker across all individuals. Most SNP loci (92%) had less than 1% missing data (see Results section). Principal component analysis (PCA) was conducted using the R statistical software package *prcomp*. To reduce dimensionality of the data, the first five principal components (PCs) were used for model-based clustering using the R package *mclust*. Here, the choice to use the first five PCs was based on the scree plot of eigenvalues [28]. The best model, which had the optimal number of clusters, was established according to the Bayesian Information Criterion (BIC), and the use of five PCs was optimal in this case. Nei's genetic distances between clusters were calculated using GenAlex [29]. The number of polymorphic SNPs within each cluster and shared between clusters was counted. For the 21 populations including the eleven fall dormancy standard check cultivars, four winter survival standard check cultivars, and five breeding populations, Nei's genetic distances were calculated using GenAlex [29]. The pair-wise matrix was used to construct an unweighted neighbor-joining tree using PHYLIP [30].

### Linkage disequilibrium

For each cluster, the LD between pairs of SNPs was estimated as r^2^ and calculated using the software program TASSEL [31]. The SNPs with minor allele frequency lower than 5% were excluded in the estimates of LD. The functional relationship between LD and physical distances on the *M. truncatula* genome was evaluated by fitting a non-linear model. The expected r^2^ value under drift-recombination equilibrium was *E*(*r* ^2^)  = 1/(1+*C*), and *C* =  4*ad*, where *d* is the physical distance in bp and *a* is an estimated regression coefficient [32]. With a low level of mutation and finite sample size *n*, the expectation becomes [33]

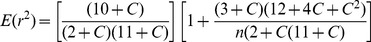



For the breeding populations BR1-4, we inferred linkage disequilibrium based on their parental genotypes. To evaluate consistency of LD between populations, we computed the correlation of LD (r_LD) between populations for SNP pairs located at distances less than 0.1 Mbp, 0.2 Mbp, or 1.0 Mbp and then plotted these correlations against the genetic distance between the pair of populations being analyzed.

## Results

### Development and validation of the SNP array

An initial set of 10,441 SNPs were selected to be included in the array, and a total of 9,277 SNPs were ultimately included. A total of 7,476 SNPs (81%) passed the quality control and showed polymorphism. The remaining SNPs either passed the quality control but were monomorphic or showed poor quality genotype clustering (Table S1). The average call rate for the 274 alfalfa genotypes was 99.6%. For the related species evaluated in this study, 97% of the 7,476 SNPs were useful for genotyping in *M. prostrata*, 91% for *M. truncatula* and *M. secundiflora*, 90% for *M. lupulina*, and 55% for *Trifolium pratense*.

Of the 7,476 SNPs, 7,433 SNPs were polymorphic among the 274 alfalfa genotypes. Of the 7,433 polymorphic SNPs, 6,826 SNPs (92%) had less than 1% missing values, 557 SNPs (7%) had 1–10% missing values, and only 50 SNPs (1%) had more than 10% missing values. Compared to diploids, up to five clusters (AAAA, AAAB, AABB, ABBB, and BBBB) could exist in tetraploid alfalfa. In total 1,025 SNPs (13.8%) showed five clearly distinguishable clusters (Table S1).

To assess the reproducibility of SNP calls, the alfalfa entry (NFAA08-258) was genotyped twice. The proportion of consistent calls for the duplicated sample was 100% (Table S3). Twenty-four samples (including 23 alfalfa genotypes and one *M. truncatula* genotype) from one SNP array chip showed a lower call rate compared to alfalfa genotypes from other chips. The four genotypes from other related species (one genotype of *M. lupulina*, one genotype of *M. secundiflora* and two genotypes of *T. pratense*) also showed lower call rates than alfalfa genotypes that were on the same chip. These 28 genotypes with lower call rates were genotyped a second time using this SNP array. The call rates for the 24 samples originating from one chip were higher in the second round of genotyping (Table S3). The average proportion of consistent calls for the 24 samples was 99.3% (Table S3). For the four samples from the closely related species, the calling rate was not significantly different and the average proportion of consistent calls was 99.9% (Table S3). Therefore, the lower calling rate for the related species was due to species differentiation rather than random technical error.

### Population structure

**Figure 1:**
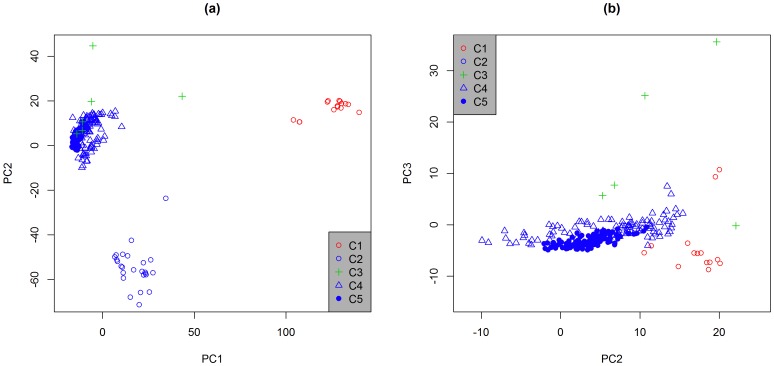
Plots of the first three principal components derived from a principal component analysis (PCA) of 263 alfalfa genotypes using SNP markers generated on an Infinium array, with (a) PC1 vs. PC2 and (b) PC2 vs. PC3. (C1: diploid subsp. *falcata*, C2: diploid subsp. *caerulea*, C3: miscellaneous tetraploid alfalfa, C4: dormant and semi-dormant tetraploid subsp. *sativa*, and C5: semi-dormant and non-dormant tetraploid subsp. *sativa*)

The principal component analysis (PCA) was performed based on 7,433 polymorphic SNP among 263 alfalfa genotypes (the F_1_ progenies were excluded). The first five principal components explained 18.2% of the total variation. The first principal component (PC1) explained 9.4% of the total variation and clearly separated diploid subsp. *falcata* from diploid subsp. *caerulea* and tetraploid alfalfa (Figure 1a). PC2 explained 3.5% of the total variation and separated diploid *caerulea* from tetraploid *sativa* (Figure 1a). Model-based cluster analysis with the first five PCs suggested five clusters of alfalfa accessions (Figures 1a and 1b). Cluster 1 (C1) consisted of the 15 diploid subsp. *falcata* genotypes; Cluster 2 (C2) contained 25 diploid subsp. *caerulea* genotypes; and Cluster 3 (C3) contained six tetraploid genotypes (one tetraploid subsp. *falcata* and five tetraploid subsp. *sativa*). Clusters 4 (C4) and 5 (C5) can be differentiated based on the distribution of genotypes from check cultivars with clearly defined fall dormancy scores. While both clusters contained about half of the intermediate dormancy (FD  = 5–7) check cultivar genotypes, C4 included 44 of 45 genotypes from the check cultivars with FD ≤4 and C5 contained 18 of 20 genotypes from check cultivars with FD 8–11 (Table 1 and Table S2). Each of these clusters included genotypes from Bulldog and the other four, non-check populations that were derived from non-dormant cultivars (Table 2). Among the five clusters, C1 was most distant from others, with an average distance of 0.119 (Table 3). The C4 and C5 clusters were most closely related (Table 3).

**Table 1:** The number of individual plants evaluated of the standard fall dormancy and winter hardiness check cultivars and the number that were assigned to either cluster 4 (C4) or cluster 5 (C5).

Fall dormancy class	Fall dormancy score	Total no. genotypes evaluated	No. genotypes in C4	No. genotypes in C5
Dormant	1–4	45	44	1
Semi-Dormant	5–7	15	7	8
Non-Dormant	8–11	20	2	18

**Table 2:** The number of individual plants evaluated for each of five breeding populations developed from non-dormant germplasm and the number of each population that were assigned to a particular cluster.

Entry	FD	Total no. genotypes evaluated	No. genotypes in C3	No. genotypes in C4	No. genotypes in C5
Bulldog 805	8	60	2	12	46
ND1	9	15		7	8
ND2	8	15		15	
ND3	9	15		1	14
ND4	10–11	15	1	1	13

**Table 3:** Pairwise genetic distances among five clusters of alfalfa genotypes based on SNP markers generated by the Infinium array.

Clusters	C2	C3	C4	C5
**C1**	0.107	0.124	0.112	0.132
**C2**		0.063	0.036	0.041
**C3**			0.022	0.024
**C4**				0.005

**Figure 2:**
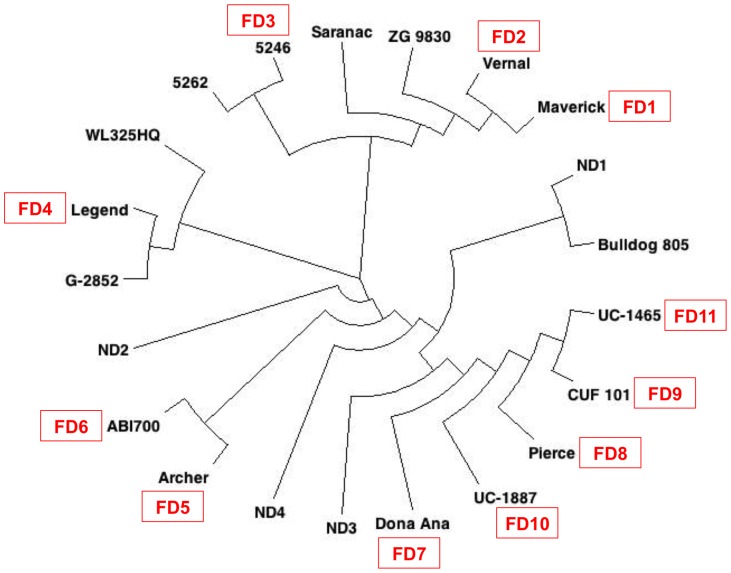
Neighbor-joining tree among 21 alfalfa cultivars and breeding populations based on SNP markers generated from an Infinium array. The fall dormancy (FD) classifications of the cultivars, which include standard check cultivars for fall dormancy and winter hardiness, are indicated on a scale of FD = 1 as very dormant to FD = 11 as very non-dormant.

Pairwise Nei's genetic distances were calculated for the 16 tetraploid standard check cultivars and five breeding populations. The neighbor-joining trees based on the pair-wise distance matrix showed that cultivars were related to one another largely based on dormancy, with cultivars more similar in dormancy being closer together on the tree (Figure 2).

### Diversity within and across sub-populations

**Figure 3:**
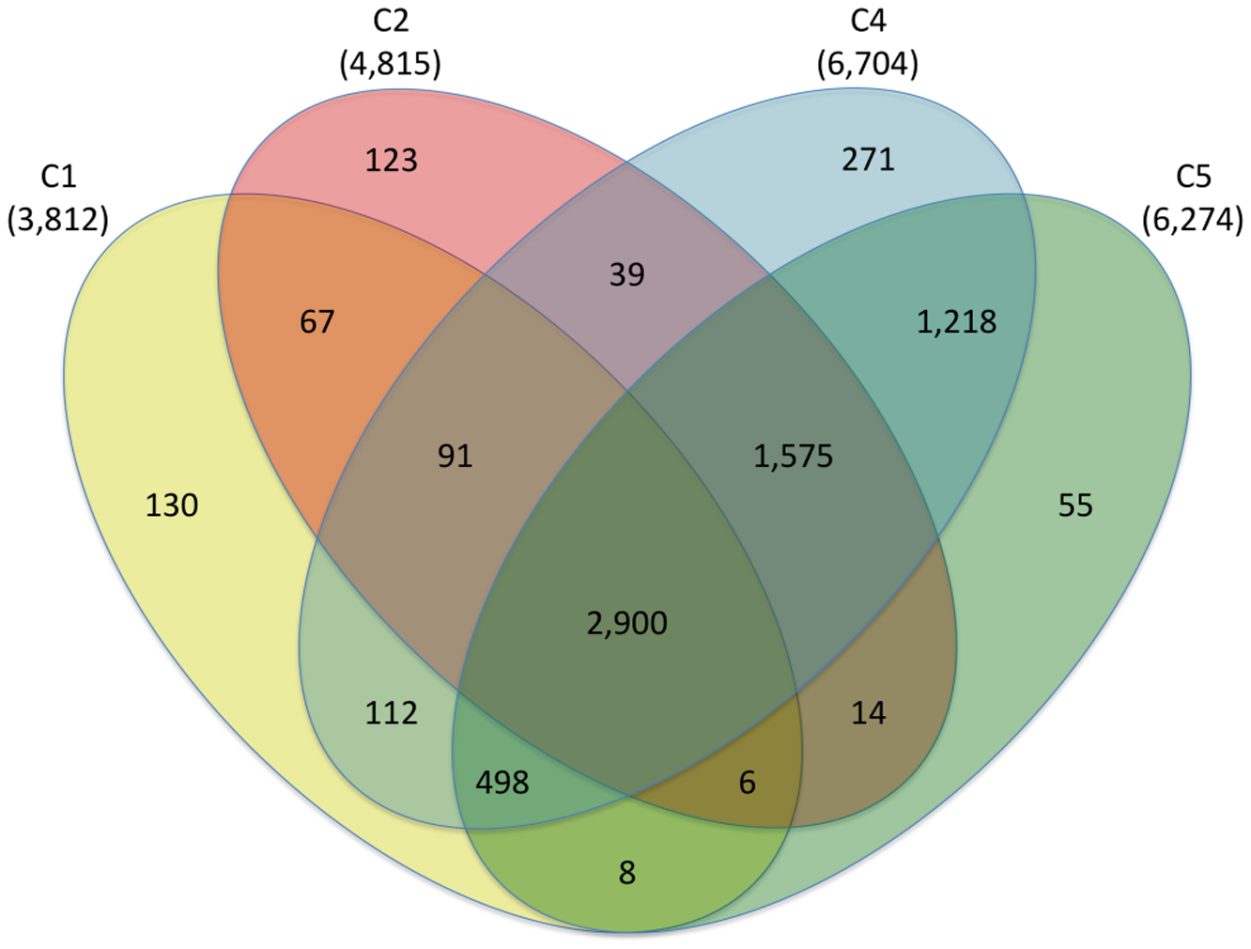
The number of polymorphic SNPs within and between four clusters developed based on a model-based clustering analysis. Cluster 3, which included only six genotypes, was omitted from the analysis.

Since C3 contained only six genotypes and its composition was not obviously related to any particular characteristic, we excluded it for the diversity analysis here. In total 7,107 SNPs were polymorphic among the remaining 257 alfalfa genotypes within C1, C2, C4, and C5. Of these, 320 SNPs (4.5%) were only polymorphic within C1 or C2 (wild diploid subsp. *caerulea* or subsp. *falcata* genotypes) but fixed in C4 and C5 (cultivated tetraploid subsp. *sativa*) (Figure 3). Of the 6,787 SNPs polymorphic within C4 or C5, most (6,191 or 91.2%) were polymorphic in both C4 and C5 (Figure 3).

Heterozygosity was calculated as the percentage of SNPs with a heterozygous call for a given genotype. The average heterozygosity was 15.8% for diploid subsp. *falcata* within the C1 cluster and 16.3% for the 25 diploid subsp. *caerulea* within C2 (Table S2). The average heterozygosity for tetraploid alfalfa genotypes was 33.8% within C4 and 33.0% within C5. For the related species, the heterozygosity level was 5.9% for *M. prostrata*, 0.8% for *M. truncatula*, 1.4% for *M. lupulina*, 1.1% for *M. secundiflora*, and 2.5% for *T. pratense* (Table S2).

### Linkage disequilibrium

**Figure 4:**
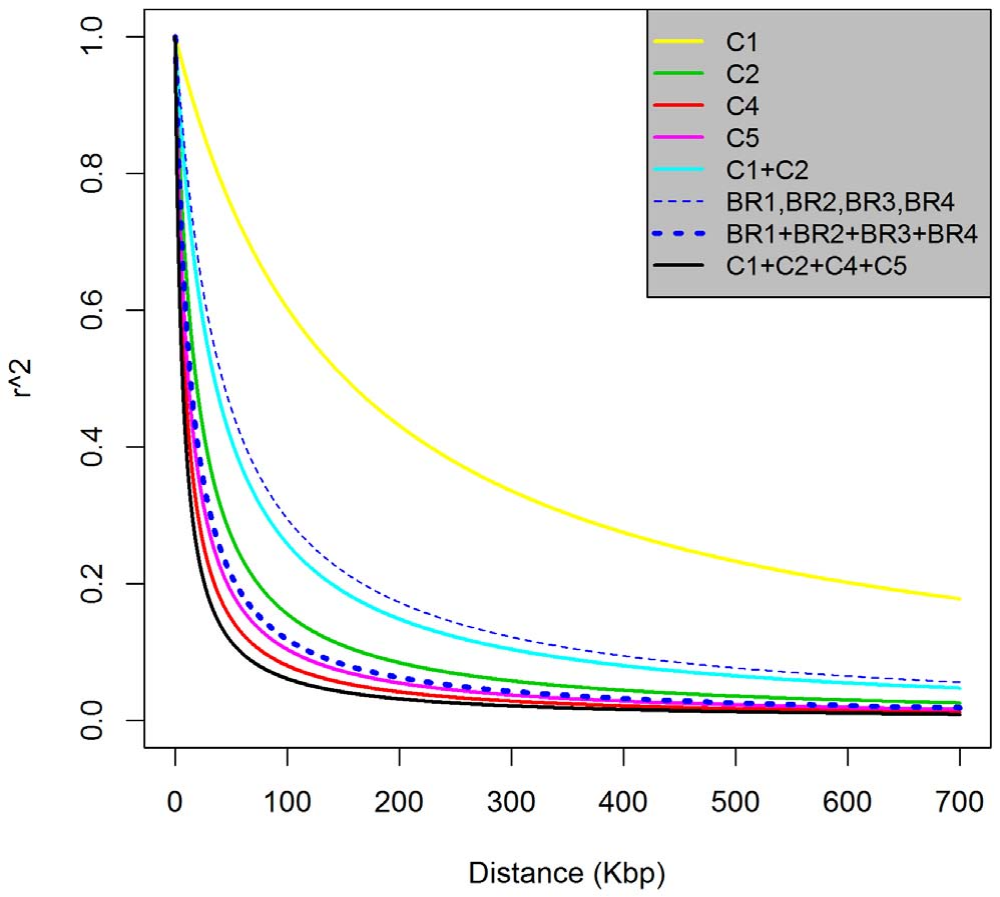
Genome-wide linkage disequilibrium patterns within clusters and populations. (C1: diploid subsp. *falcata*, C2: diploid subsp. *caerulea*, C4: dormant and semi-dormant tetraploid subsp. *sativa*, and C5: semi-dormant and non-dormant tetraploid subsp. *sativa*)

The functional relationship between LD (estimated as the square of the correlation coefficient (r^2^) between pairs of SNPs) and physical distances on the *M. truncatula* genome was determined by fitting a non-linear model. The extent of LD rapidly decayed to 26 Kbp at r^2^ = 0.2 across all the alfalfa genotypes, but varied among clusters and populations (Figure 4). The extent of LD was about 74 Kbp for C2, 35 Kbp for C4, and 47 Kbp for C5 at r^2^ = 0.2 (Figure 4). However, at r^2^ = 0.2, extensive LD was found in C1 (about 600 Kbp) (Figure 4).

For the four new breeding populations developed (BR1, BR2, BR3, and BR4), we inferred LD based on the 30 parents used to develop each of the populations. The average LD level of these four populations was expected to be about 170 Kbp at r^2^ = 0.2 (Figure 4). However, the extent of LD rapidly decayed to 54 Kbp when the four populations were combined (BR1+BR2+BR3+BR4) and analyzed as a single population (Figure 4).

### Correlation of LD across sub-populations

**Figure 5:**
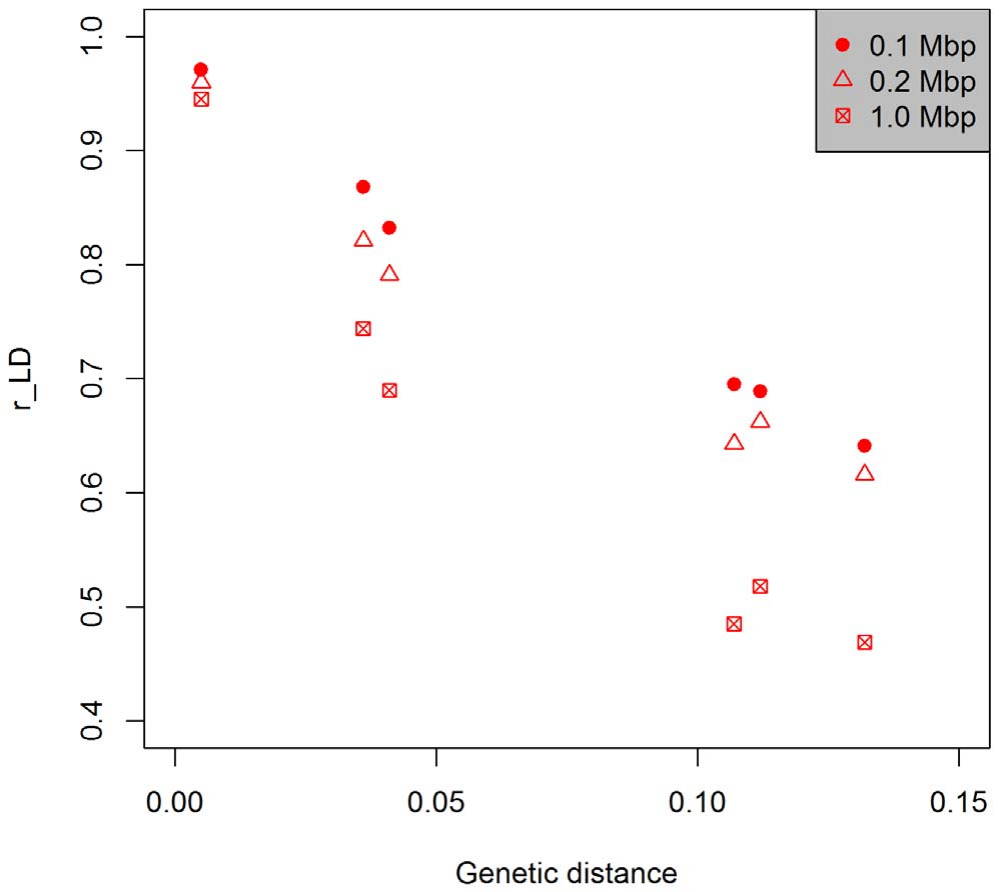
Correlation of LD values between pairs of SNP markers grouped based on three different physical distances apart and plotted as a function of genetic distances between clusters and breeding populations being correlated.

We estimated LD between pairs of SNP in different populations and then correlated these LD estimates (r_LD) between the populations. The r_LD values were negatively correlated to the genetic distance between populations (Figure 5). The LD values between pairs of SNP were more highly correlated if the SNP were physically closer together and if the populations under comparison were genetically more similar (Figure 5).

## Discussion

### Development and validation of SNP array

Illumina Infinium SNP arrays have been developed as a high-throughput genotyping platform for numerous plant species. Genotypes were called for about 72% of array features for apple [34], 70% for peach [35], 80% for sunflower [36], and 88% for tomato [37]. In this study, we developed and validated an Illumina Infinium alfalfa SNP array. In total 7,476 (81%) of the 9,274 SNPs on the array were verified and polymorphic. The successful call rate is comparable with those observed in other plant species. This array also showed a high level of transferability for closely related *Medicago* species including *M. prostrata*, *M. lupulina*, and *M. truncatula* and reasonable transferability to red clover (*T. pratense*). In addition, the allelic dosage of 1,025 SNPs (14%) could be determined in tetraploid alfalfa. In tetraploid potato, the Infinium array included 8,303 SNPs and 35% of those SNPs could be clustered into five genotypic groups [38]. Because allele dosage can affect gene expression and phenotypes in polyploids [39], [40], integrating allele dosage information into GWAS statistical models could enhance QTL identification in tetraploid species.

### Population structure and diversity

Population structure has implications for experimental design and data analysis in GWAS and GS. As in previous studies [21], [22], population structure was observed between the two subspecies of subsp. *sativa* and subsp. *falcata* in this study. In a previous experiment, no differentiation of chloroplast haplotypes was found between tetraploid subsp. *sativa* and diploid subsp. *caerulea*, which suggested that the tetraploid subsp. *sativa* simply originated from diploid subsp. *caerulea* through genome doubling [22]. Based on a small set of SSR markers, diploid subsp. *caerulea* and tetraploid subsp. *sativa* could not be distinguished from each other (Li et al., unpublished data). However, clear differentiation between them was observed using a large set of SNP markers, but only a few genotypes were analyzed [19]. In this study, we provided further supportive evidence using 263 genotypes that included wild diploid subsp. *caerulea* and cultivated tetraploid subsp. *sativa* that the two subspecies are clearly differentiated genetically. Niche differentiation may have occurred following polyploidization, early domestication, and/or modern selection in tetraploid subsp. *sativa*, and this may have caused modern alfalfa to diverge from the wild diploid progenitor subsp. *caerulea*.

Fall dormancy (FD) in alfalfa refers to the reduction of growth and the development of decumbent shoot orientation that occurs in autumn due to shortening photoperiods and/or lowering temperatures [41]. Fall dormancy is positively correlated with winter survival [42], and the alfalfa community generally uses the fall dormancy level as one criterion for choosing appropriate cultivars for planting zones. Based on crown traits, discriminant analysis of 110 cultivars showed that three classes of cultivars were identified – (1) highly dormant cultivars (FD1), (2) dormant cultivars (FD2, 3, 4, and 5), and (3) non-dormant cultivars (FD7, 8, 9); cultivars with FD  = 6 overlapped with both dormant and non-dormant classes [43]. The model-based cluster analysis generated in this study showed that C5 contained mostly genotypes from semi- and non-dormant check cultivars, and that C4 contained mostly genotypes from dormant and semi-dormant cultivars. The neighbor-joining tree of standard check cultivars suggested a gradation of relatedness based largely on dormancy level. Interestingly, the non-dormant breeding populations ND1-4 were the surviving plants selected from non-dormant germplasm following beef cattle grazing. Based on our experience, intercrossing genotypes surviving grazing pressure, as was done here, typically results in a population that is more dormant than the source population. The results here suggest that the ND1 and ND2 populations, especially, may be more dormant than their progenitor populations. Bulldog 805, itself a FD  = 8 cultivar, was selected under grazing, and some genotypes within the population fall into the more dormant cluster. Alfalfa winterhardiness has been improved by the incorporation of subsp. *falcata* germplasm, which is highly grazing tolerant, and the relationships seen among germplasm sources may reflect the amount of historical *falcata* introgression into the germplasm.

### Breeding history and population diversity

High levels of heterozygosity were observed in alfalfa, a perennial outcrossing species, while low levels of heterozygosity were found in the annual inbreeding species *M. truncatula*, or the autogamous short-lived perennials *M. lupulina* and *M. secundiflora*. The heterozygosity level of *M. prostrata* was 5.9%, which fell between that of the annual self-pollinated species and perennial outcrossing alfalfa. The perennial *Medicago* species are known to be outcrossing, although some evidence (such as low pollen to ovule ratio [44] and small flowers) suggests that *M. prostrata* may be an exception. Our results indicate that *M. prostrata* likely outbreeds to some extent in the wild. *M. prostrata* can easily be crossed with alfalfa and used to improve pest resistance [45].

Minimal diversity appeared to have been lost in cultivated tetraploid alfalfa compared to wild diploid alfalfa based on a transcriptome analysis of 27 genotypes [19]. In this study, we genotyped a larger population of individuals with the SNP array and found a similar result. Our results are in contrast to a previous experiment that showed that domesticated alfalfa contained on average 31% less diversity than wild alfalfa [46]. The different results could be due to the different populations being evaluated, the marker types used, and the number of markers. Nine major alfalfa germplasms have been introduced into the US historically [47]. However, many new landraces and plant introductions, including even wild diploid germplasms, have been subsequently continuously integrated into elite breeding germplasms [48]. This germplasm introduction process, together with a relatively short breeding history and the outcrossing and polyploid nature of alfalfa, could explain the high diversity retained in cultivated tetraploid alfalfa. However, ascertainment bias could partially explain the results, because the SNPs used for the array were primarily derived from cultivated tetraploid alfalfa sequences.

More than 90% of the SNPs polymorphic in tetraploid alfalfa were polymorphic in both cultivated alfalfa sub-populations. Multiple germplasms have been mixed in most breeding programs since the 1950's [47] and both dormant and non-dormant cultivars have often included germplasm from many of the historical introduction groups in their parentage [49]. However, the major historically important germplasms contributing to the dormant and non-dormant alfalfa gene pools are different. For example, the top five germplasms contributing to current dormant cultivars are Flemish (39%), *varia* (22%), Turkistan (12%), Ladak (8%), and Chilean (7%) [49]. The top four major germplasm sources for non-dormant cultivars are African (34%), Indian (17%), Turkistan (14%), and Chilean (9%) [49]. Therefore, although a broad differentiation in the background of dormant and nondormant germplasm exists, recombination during breeding has made this distinction less clear, and consequently, our clustering results reflect this pattern.

### Patterns of linkage disequilibrium

Linkage disequilibrium is a key factor in determining the number of markers needed for GWAS and GS. Using the Infinium alfalfa SNP array, we evaluated the genome wide LD pattern for the first time using a large set of markers for alfalfa. Overall, LD decayed to an r^2^ = 0.20 within 26 Kbp, but this varied greatly among sub-populations. We estimated LD to be about 170 Kbp at r^2^ = 0.2 within four newly developed breeding populations (BR1, BR2, BR3, and BR4), each generated from 30 parents. However, LD rapidly decayed within 54 Kbp when we evaluated all 120 parents together. Populations with fewer founders will require a lower marker density for GWAS or GS, but using populations of this sort may result in inbreeding depression, which can affect results and needs to be carefully considered [50]. Nevertheless, we argue that alfalfa breeding programs will need to move to smaller populations to fully realize efficiency gains from genetic marker resources.

Many factors such as recombination, mutation, genetic drift, selection, and population admixture affect the extent of LD. If we analyzed diploid subsp. *caerulea* and subsp. *falcata* together, LD increased, possibly due to the divergent genomes generating extensive admixture LD. Similarly, extensive admixture LD was found in barley when divergent materials were mixed for LD evaluation [51]. The more extensive LD that we found within the BR1/2/3/4 breeding populations (of 30 parental genotypes each) compared to the cluster of 25 wild subsp. *caerulea* genotypes indicates that by selecting subsets of genotypes from modern cultivars, breeders can increase LD to acceptable levels for genome-wide marker-based breeding projects.

Successful GWAS and GS experiments also rely on the consistency of LD within the employed population. Natural and/or artificial selection that occurred in wild and breeding populations may result in different allelic combinations and in phasing across loci. Marker-trait association in GWAS and estimated marker effects in GS could be biased or obscured when divergent populations are combined [51]. We identified a high level of consistency in LD between two large subpopulations of cultivated alfalfa in this study. However, wild diploid alfalfa, especially diploid subsp. *falcata*, is divergent from cultivated tetraploid alfalfa and showed a low level of LD consistency at a distance of 0.1 Mbp. Therefore, it is not advisable to combine those germplasms for GWAS and GS.

This experiment reports on the development and testing of an Illumina Infinium array for alfalfa. The array can assay nearly 8,000 SNP markers simultaneously, providing the community with the most advanced genotyping platform currently available. It should prove useful not only for genetic diversity experiments but also for genetic mapping, GWAS, and possibly GS. The array is available to any researcher and can be obtained commercially from Illumina, Inc. (www.illumina.com).

## Supporting Information

Table S1 **Characteristics of the 10,441 SNPs selected for the alfalfa Illumina Infinium SNP array.**
(XLSX)

Table S2 **Accessions used for evaluation of the alfalfa Illumina Infinium SNP array.**
(XLSX)

Table S3 **Call rates of the duplicated and repeated samples.**
(XLSX)
